# TUBERCULOSIS OF THE CHEST WALL WITHOUT PULMONARY INVOLVEMENT

**DOI:** 10.4103/0970-2113.59597

**Published:** 2008

**Authors:** G.S. Gaude, AK Reyas

**Affiliations:** 1 Professor and Head Department of Pulmonary Medicine, J.N. Medical College, Belgaum, Karnataka, India; 2 Post Graduate Student Department of Pulmonary Medicine, J.N. Medical College, Belgaum, Karnataka, India

**Keywords:** tuberculosis, chest wall

## Abstract

Skeletal tuberculosis is usually seen in association with primary pulmonary form. Pulmonary tuberculosis of the chest wall is a rare entity. We herein report a case of tuberculosis of the chest wall without pulmonary involvement that presented with big ulcer in the anterior chest wall and responded completely to the antituberculosis therapy without any surgical intervention.

## INTRODUCTION

Tuberculosis (TB) has become a significant international public health problem, especially in the developing countries largely because of the widespread immigration, malnutrition and due to the widespread HIV infection^1^. Chest wall tuberculosis is rare and still a diagnostic and therapeutic challenge. Cold abscess, meaning swelling without inflammation, is the characteristic presentation of the chest wall TB^2^. It can present as an isolated lesion without any primary foci in the lung parenchyma or in the ribs. We hereby present a case of tuberculous ulcer over the chest wall as the initial presentation without any lesion in the lungs or in the bones including ribs.

## CASE REPORT

A 26 year old male, A.P. presented to the chest clinic with a painless nodular swelling over the left lower chest in the midclavicular region. This nodular swelling was present for 3 months prior to the presentation and it gradually increased over a period of 2 months, and then it became soft and burst open one day with thick serosanguinous pus. Over a period of next one month, the discharge from this open ulcer continued and it became bigger and also extended deep into the skin involving the subcutaneous tissue. This ulcer was painless and had produced a big defect in the chest wall. He was being treated with local applications of antibiotics and oral antibiotics without much relief. There was history of intermittent fever initially for about 15 days. There were no respiratory complaints and no history of anorexia or weight loss. There was no history of tuberculosis either in childhood or adulthood. He was a nondiabetic and nonalcoholic.

**Fig. 1:**
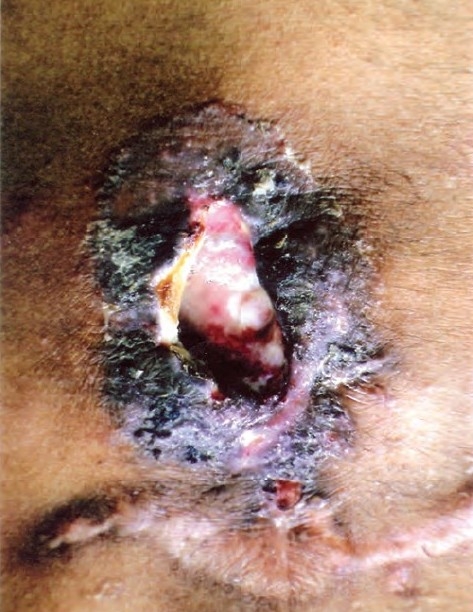
Large oval ulcer over left lower chest 6.5 × 4.5 cms with undermined edges having yellowish slough and serous discharge.

**Fig. 2:**
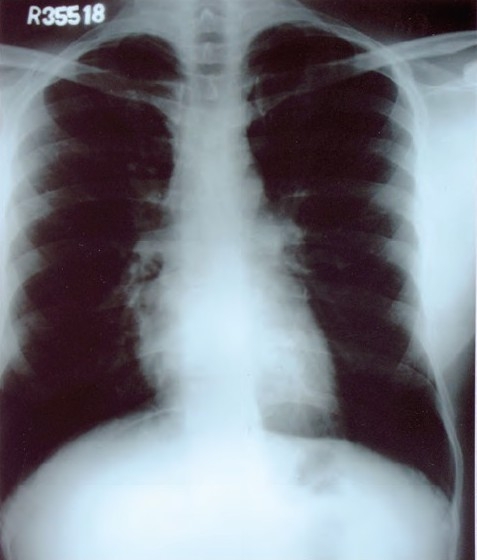
Normal chest radiograph film without any mediastinal lymphadenopathy.

**Fig. 3:**
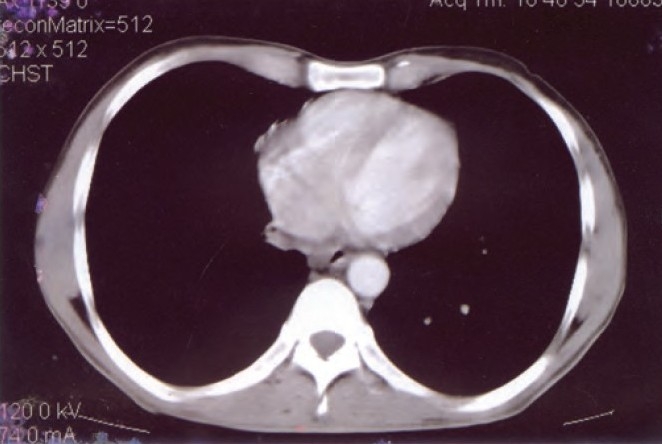
CT Scan of the thorax showing no abnormality in the lung parenchyma and no mediastinal lymphadenopathy.

**Fig. 4:**
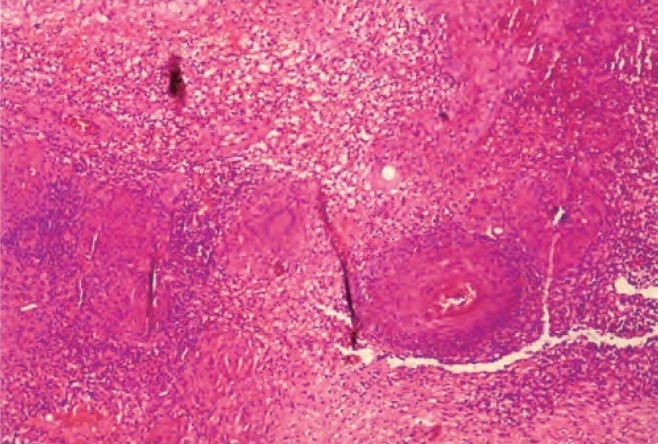
Biopsy from the ulcer edges shows skin tissue showing granulomas consisting of epitheliod cells, Langhan's type of giant cells, lymphocytes and macrophages.

Examination showed averagely built male with BMI of 23.5, no clubbing and lymph adenopathy. Respiratory system examination was normal. Local examination revealed a large solitary oval ulcer over left lower chest in 6th intercoastal space in the midclavicular region of 6.5cms X 4.5 cm in dimensions, with undermined edges, having yellowish slough with scanty serous discharge (Fig. 1). The ulcer was extending deep into the skin almost to an extent of 2.5cms involving subcutaneous tissue with raised edges and had vegetations and crusts; it was slightly tender, and surrounding skin was indurated. There was no involvement of the regional lymph nodes. Tuberculosis was kept in mind in investigating this ulcer as it was progressing over a period of 3 months. His hemogram was normal, HIV and VDRL were negative. Chest radiograph was normal with no evidence of hilar adenopathy (Fig. 2). CT Scan of the thorax was also performed, which revealed no parenchymal infiltrations or cavity, and also there was no evidence of any mediastinal lymphadenopathy (Fig. 3). PPD test was 16 mm. Biopsy from the edge of the ulcer was done which showed granulomas consisting of epitheloid cells, Langhans' type of giant cells, lymphocytes and macrophages (Fig. 4). Fungal elements were negative. The findings were suggestive of tuberculous ulcer. As the diagnosis of tuberculosis of the chest wall was made, and there were no primary involvement of the lungs, every effort was made to find out any primary lesions anywhere in the body. This included examination of the oral cavity, ears, skin, conjunctiva, abdomen, kidneys and the bones. Abdominal ultrasonography did not revealed any mass or cystic lesions in the intestines or in the kidneys. But no primary lesion anywhere in the body could be identified.

**Fig. 5:**
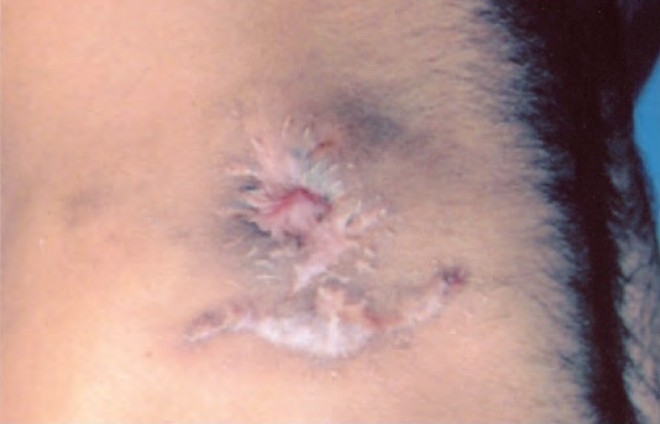
Tuberculous ulcer at the end of the therapy without any surgical intervention. It has healed up completely with no defect left behind.

Thus, the diagnosis of primary chest wall tuberculous ulcer was made and he was started on autituberculosis regimen -Category III regimen with Isoniazid, Rifampicin and Pyrazinamide thrice weekly under RNTCP. After one month of therapy, the ulcer started regressing in size, slough disappeared and serous discharged stopped. After the end of 6 months of therapy, the ulcer healed completely and the opening in the chest wall closed completely (Fig. 5). He was given extended therapy of Isoniazid and Rifampicin for another 2 months to prevent the recurrence. He is doing well during the follow up period.

## DISCUSSION

Tuberculosis of the chest wall constitutes 1% to 5% of all cases of musculoskeletol TB, which in turn is far less frequently encountered than pulmonary infection alone and represents between 1% to 2% of TB overall^3^. Tuberculous abscess of the chest wall can involve the sternum, costochondral junctions, rib shafts, costovertebral joints and the vertebrae. They are most frequently found at the margins of the sternum and along the rib shafts^4^. Multiple lesions over the chest wall can also be observed in half of the cases; mainly due to the suppressed immunological response by the host tissue.

Our case had indolent ulcer over the chest wall with corrugated margins and lot of granulation tissue, and purulent greenish discharge from the edges. There was neither any evidence of primary focus in the lungs nor any mediastinal lymphadenopathy. This suggests that chest wall involvement was of primary in nature. There was also no rib destruction adjacent to the ulcer. More then half of the lesions of rib tuberculosis shows no evidence of rib destruction^2^. Lee^5^ reported rib destruction in 69% of the cases. While in another study, CT scan of thorax could diagnose rib involvement in only one case^6^. The association of a soft tissue mass, osteolytic lesion and sequestrum suggests chest wall tuberculosis on CT of thorax.

Chest wall tuberculosis may occur by means of two mechanisms: (i) hematogenous dissemination associated with activation of a dormant tuberculous focus, and (ii) direct extension from a lymphadenitis of chest wall^7^. Burke^8^ described the steps of the evolution of cold abscess of chest wall with the aid of his well designed experimental and anatomical studies as follows: tuberculosis bacilli invade the pleural space and set up local or widespread pleuritis; some bacilli transported from pleural space to parasternal (or posterior intercostal) lymph nodes; there nodes become caseous and rupture; necrotic and caseous material burrows anteriorly (or posteriorly) to form a cold abscess in the chest wall.

Our patient had already big 6.5cm × 4.5cm indolent ulcer at the time of presentation in the midclavicular location. He had passed through the phases of cold abscess and sinus formation. It was misdiagnosed earlier as pyogenic abscess. Kuzucu^2^ et al reported 6 cases of chest wall tuberculosis who presented as cold abscesses with an average diameter of 7.8 cm and one patient was having draining sinus. The diagnosis of chest wall tuberculosis has to be based on bacteriological or histological confirmation as it is true in all tuberculosis cases. In our case diagnosis was established by the biopsy taken from the edges of the ulcer.

The treatment of chest wall tuberculosis is controversial. There are some series^9^ reporting good results with only antituberculosis drugs. But in the other series^10^, abscesses were not cured and even recurred or progressed despite adequate medical treatment. In our case only anti tuberculosis drugs were given and his ulcer headed completely without any surgical intervention. If medical treatment is not sufficient then these cases requires wide debridement along with anti- tuberculosis medications. In a large series of 89 patients with chest wall abscess due to TB, Paik et al^11^ performed excision of abscess in 28% and excision of abscess and rib in 72% of the patients. They recommended preoperative and post operative anti-TB drugs and complete resection of chest wall mass including any suspicious ribs. Although WHO recommends a standard 6-month regimen, according to clinical presentation, bacillary load and response to an antibiotherapy, the treatment can be extended up to 9-12 months^2^.
